# Interventions to Drive Improved Functional Potential of Aged or Dysfunctional Hematopoietic Stem Cells

**DOI:** 10.1093/geroni/igaa057.2665

**Published:** 2020-12-16

**Authors:** Isabel Beerman, Le Zong, Mayuri Tanaka-Yano, Hagai Yanai, Ferda Tekan-Turhan

**Affiliations:** 1 NIA, Baltimore, Maryland, United States; 2 NIA / NIH, Baltimore, Maryland, United States; 3 NIA/ NIH, Baltimore, Maryland, United States

## Abstract

Stem cell dysfunction is a hallmark of aging, associated with the decline of physical and cognitive abilities of humans and other mammals. Therefore, it has become an active area of research within the aging and stem cell fields, and various techniques have been employed to mitigate the decline of stem cell function both in vitro and in vivo. We have examined changes in the hematopoietic system after interventions and show modest, but positive effects on the aged system as well as the aged stem cells

